# Validation of MLC‐based linac radiosurgery for trigeminal neuralgia

**DOI:** 10.1002/acm2.12381

**Published:** 2018-06-14

**Authors:** M. Tynan R. Stevens, Eric C. Lobb, Kamil M. Yenice

**Affiliations:** ^1^ Department of Radiation and Cellular Oncology University of Chicago Medicine Chicago IL USA

**Keywords:** dosimetry, film, multi‐leaf collimator, radiosurgery, scintillator, trigeminal neuralgia

## Abstract

This study details a validation process for linear accelerator‐based treatment of trigeminal neuralgia using HD‐MLC field collimation. Nine trigeminal neuralgia treatment plans utilizing HD‐MLC were selected for absolute dose measurement at isocenter using a commercial scintillating detector in an anthropomorphic phantom. Four plans were chosen for film dosimetry measurements in each of the three principal planes to assess spatial dose distribution agreement with the treatment planning system. Additionally, trajectory log analysis for each treatment field in the nine cases was performed to assess mechanical positioning accuracy of the MLC system during delivery. Scintillator and film measurements both revealed mean dose agreement at isocenter of better than 3% while FWHM of the 2D dose distribution in each plane showed agreement between plan and measurement within 0.2 mm. Analysis of log files revealed a maximum MLC leaf positioning error of 0.04 mm across 178 treatment fields. In conjunction with a quality‐controlled treatment delivery methodology, an appropriately commissioned treatment planning system can be used for accurate and clinically appropriate design of trigeminal neuralgia treatment plans utilizing HD‐MLC.

## 1 INTRODUCTION

After more than five decades of development, stereotactic radiosurgery (SRS) is a widely available treatment technique for intracranial tumors using both dedicated units like Gamma Knife and CyberKnife, as well as linear accelerators (linacs).^1^ SRS uses finely collimated beams to deliver ablative doses in a single or few fractions to treat benign and malignant tumors in the brain. SRS delivery on linacs became possible only after refinements in mechanical precision of the delivery system. Early investigators of linacs as a radiosurgery tool constructed special hardware that attached to the accelerator and allowed a higher degree of precision to be achieved than was possible with standard linac treatments.^2^ The use of a tertiary collimating system with circular apertures closer to the patient on a linac reduces both the beam penumbra and the susceptibility to positioning error.^3^ Modern linacs for SRS delivery from vendors come with both circular collimator attachments and a computer‐controlled multi‐leaf collimating (MLC) system which is integrated in the head of the machine to shape the beam. The user has the option of using either beam shaping device depending on the application. Further refinement in MLC technology has resulted in MLC systems with finer leaf widths (2.5–3.0 mm) for the purpose of treating very small targets.^4^, ^5^ With the improved technology, modern machines can achieve overall couch/gantry/collimator isocentric accuracy within 0.6 mm.^6^ When these machines are combined with image guidance capabilities using cone‐beam CT (CBCT) or in‐room kV imaging (ExacTrac), a targeting accuracy of 0.5 ± 0.2 mm can be achieved for small intracranial targets.^7^ Users of modern linacs as a SRS tool have unprecedented choices of various techniques for the treatment of radiosurgery targets.

A number of groups showed the efficacy of static conformal arc, dynamic conformal arc, or intensity‐modulated treatments with MLC delivery to treat various indications including acoustic schwannoma, arteriovenous malformation, meningioma, and metastasis.^8^, ^9^ Treatment of trigeminal neuralgia with linac SRS appears to be limited to the use of arcs with circular shaped collimators.^10^ To our knowledge, there is no study of MLC‐based SRS for trigeminal neuralgia reported in the literature. The purpose of this paper is to demonstrate the validity and reliability of HDMLC SRS delivery for trigeminal cases. In this paper we report results for: (a) verification of MLC beam shapes, (b) total dose delivered to representative treatment volume (a plastic scintillator), and (c) the shape of the cumulative dose distribution (via film).

## 2 MATERIALS AND METHODS

### 2.A Case details

**Figure 1:**
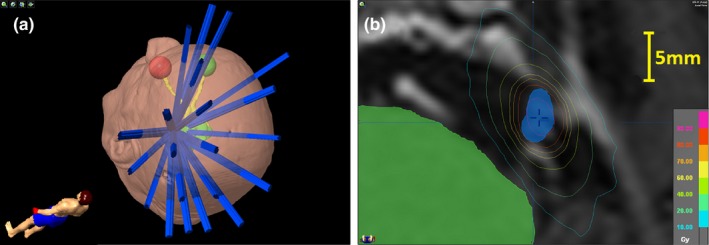
Example of field distribution for trigeminal neuralgia case (a) along with target volume and axial isodose distribution displayed on T1‐weighted MRI (b). The trigeminal nerve target volume is in blue while the brainstem is shown in green. The displayed isodose lines range from 10 Gy (cyan) to 90 Gy (pink).

Nine clinical trigeminal neuralgia treatment plans were selected for verification testing. These consisted of a mix of left‐sided and right‐sided cases, including one patient with bilateral target volumes. The prescribed dose ranged from 6000 cGy for the bilateral case up to 8750–9375 cGy for all other cases. Target volumes ranged in size from 0.026 to 0.068 cc (mean value 0.041 cc) with per‐plan equivalent square field size ranges of 0.59–0.70 cm (mean value 0.67 cm). For each plan, the minimum per‐field equivalent square field size was restricted to 0.50 cm, the smallest field size for which output factors and beam profiles were directly measured during TPS commissioning. An example of a trigeminal neuralgia target volume and associated treatment field distribution and isodose distribution near the mean of the group is shown in Fig. 1.

All treatment plans utilized the 6 MV FFF treatment beam and were planned for delivery on a Varian Truebeam STx linear accelerator (Varian Medical Systems, Inc., Palo Alto, CA) equipped with HDMLC (0.25 cm leaf width projection at isocenter) and ExacTrac with 6D robotic couch (Brainlab AG, Munich, Germany). Plans were delivered with 18–21 static conformal fields (Table 1), depending on the patient geometry and required dose fall‐off. All plans were remapped for phantom delivery from clinically accepted patient treatment plans. For this study, all identified cases had an absolute dose measurement performed at isocenter along with aggregated analysis of trajectory log files. Four cases representing the range of field sizes observed were also selected for film dosimetry verification.

**Table 1:** Per‐case absolute dose results comparing iPlan computed dose (*D*_TPS_) with dose measured using W1 scintillator detector (*D*_measured_). Mean equivalent square field size (FS) for each plan is also provided

Case	# Fields	Mean FS (mm)	*D*_TPS_ (cGy)	*D*_measured_ (cGy)	Difference (%)
1	21	6.3 ± 0.7	9111	9603	−0.5
2	21	7.7 ± 0.4	9642	9746	1.1
3	18	5.9 ± 0.5	6311	6137	−2.8
4	18	6.2 ± 0.4	6472	6324	−2.3
5	20	6.9 ± 0.4	9658	9658	0.0
6	20	7.1 ± 0.4	9231	9292	0.7
7	19	6.8 ± 0.2	9404	9349	−0.6
8	20	6.7 ± 0.4	9507	9535	0.3
9	21	7.0 ± 0.3	10,501	10,531	0.3
Mean	20	6.7 ± 0.7	–	–	−0.4 ± 1.2

### 2.B Mechanical delivery precision

TrueBeam trajectory log files were collected during delivery of the nine clinical plans to assess the mechanical precision of the MLCs during treatment delivery. The log files include the planned and recorded position of each MLC leaf throughout the treatment delivery. As static fields were used for delivery, the MLC positions in the log files are constant throughout each field, with the X and Y collimator jaws automatically set to 2–3 mm behind the MLC opening during the planning process. The error in the leaf positions was thus calculated as the difference between the expected and actual values for the first control point in each field. Only those leafs that were involved in defining the field aperture were included in the analysis, resulting in 1270 total samples across all clinical cases. This test specifically does not include the effects of gantry sag and finite isocenter accuracy, which is monitored via day‐of‐treatment Winston‐Lutz tests delivered to the portal imager using MLC‐defined fields. These sag and walkout effects are however pertinent to the absolute dose and dose distribution tests described below. An institutional tolerance of 0.5 mm on the daily Winston‐Lutz test is used routinely.

### 2.C End‐to‐end testing

#### 2.C.1 Absolute dose verification

Cumulative absolute dose was verified at isocenter for each of the nine clinical cases. Verification measurements were performed within a CIRS Model 605 (CIRS, Inc., Norfolk, VA) anthropomorphic head phantom (“BRUNO”) using the Exradin W1 (Standard Imaging, Inc., Middleton, WI), a commercial plastic scintillating fiber‐based detector. The scintillating fiber of the W1 has a diameter of 1 mm and a length of 3 mm and is minimally perturbing to the incident radiation field due to its near water‐equivalent construction.^11^ The W1 was cross‐calibrated with an ADCL‐calibrated farmer chamber (Exradin A12, Standard Imaging, Inc.).

**Figure 2:**
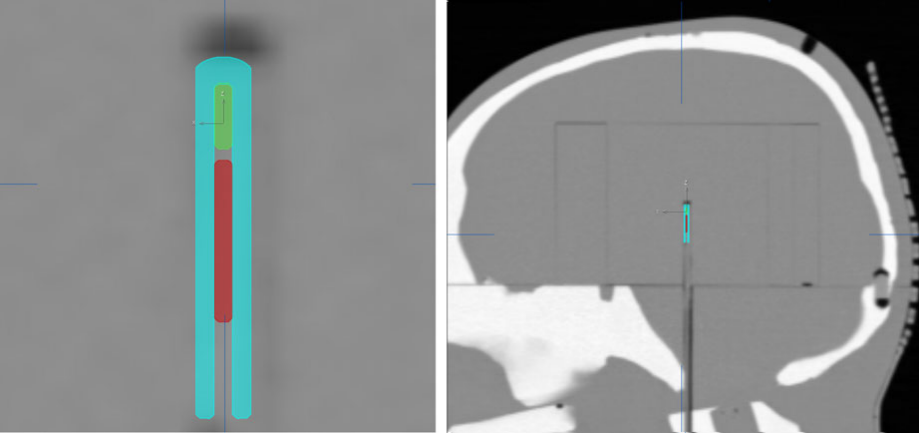
Model of W1 detector within the BRUNO CT dataset. The ABS shell is shown in teal, the fluorescent‐doped polystyrene scintillating fiber is shown in green, and the PMMA optical fiber core is shown in red.

The BRUNO phantom underwent CT simulation in which a Brainlab mask was constructed for reproducibility of phantom positioning. Axial slices through the entire length of the phantom were acquired at 1 mm thickness for patient plan mapping while 0.5 mm slices were acquired through the extent of the W1 block insert. The two scans were registered within the iPlan treatment planning system and the 0.5 mm images were used for accurate geometric reconstruction of the W1 to facilitate accurate density modeling and dose prediction (Fig. 2).

Clinical treatment plans were mapped to the BRUNO dataset with isocenter placed in the centroid of the scintillating fiber. All table, collimator, and gantry angles were left intact from the clinical treatment plan. After 3D dose computation, plan‐specific adjustments to isocenter position were introduced to best center the W1 within the dose cloud such that variation in dose across the fiber was minimized. The magnitude of these isocenter adjustments ranged from 0.0 to 0.8 mm. Dose was recomputed following these fine adjustments, and mean dose to the scintillating fiber structure was recorded as the planned dose value for comparison against measurement.

The scintillator response used in conjunction with the SuperMAX two‐channel electrometer (Standard Imaging, Inc.) was calibrated immediately prior to performance of these verification measurements using the methodology recommended by the vendor, which is based on work by a number of researchers.^12^, ^13^, ^14^, ^15^ The primary goal of this calibration is to correct for Cerenkov emissions created proportional to the irradiated length of the optical fiber. The calibration process consists of performing large‐field irradiations of the W1 with minimum and maximum lengths of the optical fiber within the radiation field and computing a Cerenkov light ratio (CLR) correction according to eq. (1):

(1) $$CLR=\left(SC1_{\mathrm{MAX}}-SC1_{\mathrm{MIN}}\right)/\left(SC2_{\mathrm{MAX}}-SC2_{\mathrm{MIN}}\right)$$

SC1 and SC2 refer to the charge reading from the blue and green spectral regions of the photodiode while the subscripts MIN and MAX refer to the amount of optical fiber within the radiation field. The gain of the scintillator is further adjusted such that the electrometer reads out directly to dose by application of eq. (2):

(2) $$Gain=Dose_{10}/\left(SC1_{MIN,10}-SC2_{MIN,10}\times CLR\right)$$

The additional subscript “10” in the above equation refers to the fact that the calibration and absolute dose is based on the delivery of a known dose using a 10 × 10 cm field in the minimum fiber configuration. This calibration formalism is applied in general to dose measurements according to eq. (3):

(3) $$Dose=Gain\times \left(SC1-SC2\times CLR\right)$$

For treatment delivery the BRUNO phantom was immobilized using the Brainlab mask system constructed during CT simulation. Six‐dimensional CBCT‐based corrections to the phantom position were implemented using the ExacTrac robotic couch in order to match the phantom position with the CT dataset used in the verification plan, including the previously mentioned sub‐millimeter adjustments to isocenter position.

All treatment fields were delivered at the clinical gantry, collimator, and couch rotational positions. For the W1 measurements there were no additional corrections to the phantom position made after couch rotations, such as through the use of the ExacTrac kV x‐ray system. Couch walkout for this particular treatment machine is tested on a monthly basis and has a value of less than 0.6 mm across the full range of couch motion, expressed as the maximum distance between beam center intersections for a couch star‐shot film.

Per‐field and per‐plan doses were tabulated and compared to iPlan calculation for each of the 178 treatment fields spread across the nine patient plans.

#### 2.C.2 3D dose distribution verification

Film measurements were performed to evaluate spatial dose deposition accuracy of the full treatment delivery. The BRUNO phantom has a bisected insert which can accommodate an approximately 6.3 cm^2^ piece of GafChromic EBT3 radiochromic film (Ashland Specialty Ingredients, Bridgewater, NJ). The insert can be oriented within the phantom to place the film in any of the three perpendicular planes. The BRUNO phantom underwent additional CT scans at 1 mm slice thickness with the film oriented in the transverse, sagittal, and coronal planes and the scans were evaluated to verify that there was no yaw, pitch, or roll which would result in the film having a skewed or out‐of‐plane orientation.

Pre‐cut films provided by the manufacturer were used for all film measurements, and all films came from the same lot. Films were scanned using an Epson 10000XL scanner at 300 DPI and 48 bit color. Time between irradiation and scanning was 24 ± 1 h, during which films were kept in light‐proof containers. A film holder template was fabricated to ensure consistent position and orientation on the scanner bed for each film.

##### Film calibration

Film calibration and readout was performed following methods consistent with previous published recommendations.^16^, ^17^, ^18^, ^19^ Calibration films were irradiated using 3 × 3 cm^2^ MLC defined fields, with the jaws set to 4 × 4 cm^2^. Films were irradiated at 95 cm SSD, 5 cm depth in solid water. Dose to isocenter in this geometry was calculated as 0.938 cGy per monitor unit. Calibration exposures of 0, 100, 200, 250, 300, 350, 400, 450, 500, and 600 MU were obtained, corresponding to a dose range of 0–5.6 Gy. Each film was marked for consistent orientation and numbered upon removal from the package. Scanned films were read into the RIT film dosimetry system (Radiological Imaging Technology, Inc. Colorado Springs, CO), and the central 1 × 1 cm^2^ area was averaged to produce dose calibration points, with the resulting curve interpolated using a polynomial fit.

##### Film measurement

Four cases spanning the range of mean field sizes were selected for film analysis. The film insert contains a central low‐contrast sphere which was contoured and used for isocenter placement of the mapped treatment group within the iPlan software. Prior to dose calculation the prescription was scaled down to a maximum isocenter dose of 500 cGy in order to facilitate more accurate film calibration over a narrower range. Two‐dimensional dose distributions centered on isocenter were exported in DICOM‐RT format through the transverse, coronal, and sagittal planes.

Prior to treatment delivery the BRUNO phantom with film insert was positioned using the ExacTrac kV x‐ray system. Imaging was repeated after each couch rotation to ensure that couch walkout did not appreciably affect the phantom position. Using a technique of 80 kVp and 10 mAs per image, the total imaging dose contribution according to previous measurements is estimated at approximately 1 mGy, and can safely be ignored.^20^ Positioning tolerance with the ExacTrac x‐ray system was set to 0.5 mm and 0.5°, and any verification imaging that suggested corrections larger than these values resulted in 6D corrections being applied followed by repeat verification imaging. Each treatment field was delivered at the clinical couch, collimator, and gantry positions and the process was performed a total of three times, once for each film orientation.

Scanned films were read into RIT for analysis, and the dose calibration curve was applied. A 5 × 5 median filter was applied to the scanned images for noise reduction. The FWHM of the dose distribution was determined in all three principal axes from one‐dimensional profiles passing through the maximum dose point of each film. The exported dose planes were read into RIT as reference images, and the FWHM of the planned dose was likewise obtained. The planned and measured dose planes were co‐registered, and gamma analysis of both the 2D planes and 1D profiles through isocenter using a global 3%/1 mm criteria was performed. 2D isodose overlays were generated and inspected visually to further establish agreement between the planned and delivered dose distributions.

## 3 RESULTS AND DISCUSSION

### 3.A Mechanical delivery precision

**Figure 3:**
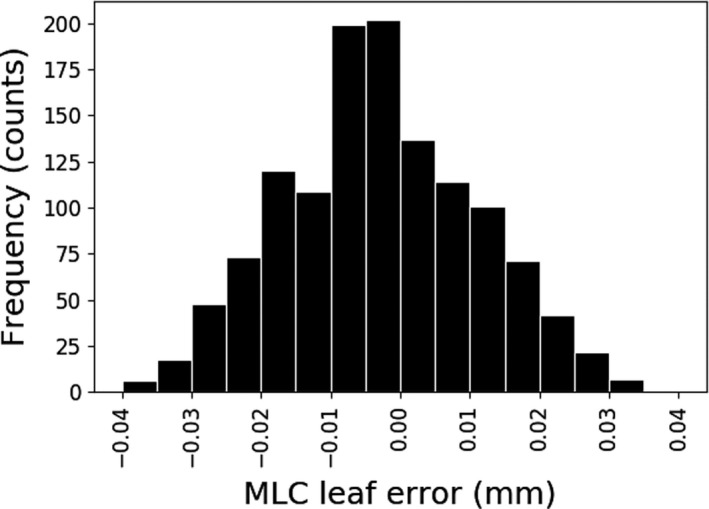
Distribution of MLC leaf position errors for all fields.

The distribution of MLC leaf deviations from their planned positions is shown in Fig. 3. The mean error magnitude is 0.01 mm, with no observed bias toward MLCs being open more or less than in the plan. The maximum error magnitude observed was 0.04 mm. These observed errors are consistent with our monthly EPID‐based MLC quality assurance results, which demonstrate median errors in a picket fence delivery of 0.02–0.04 mm. It should be noted that these results are based on a static MLC delivery scheme. In the case of a dynamic delivery technique it would be expected that somewhat larger differences would be observed and would need to be evaluated in the context of overall delivery accuracy.

### 3.B End‐to‐end testing

#### 3.B.1 Absolute dose verification

**Figure 4:**
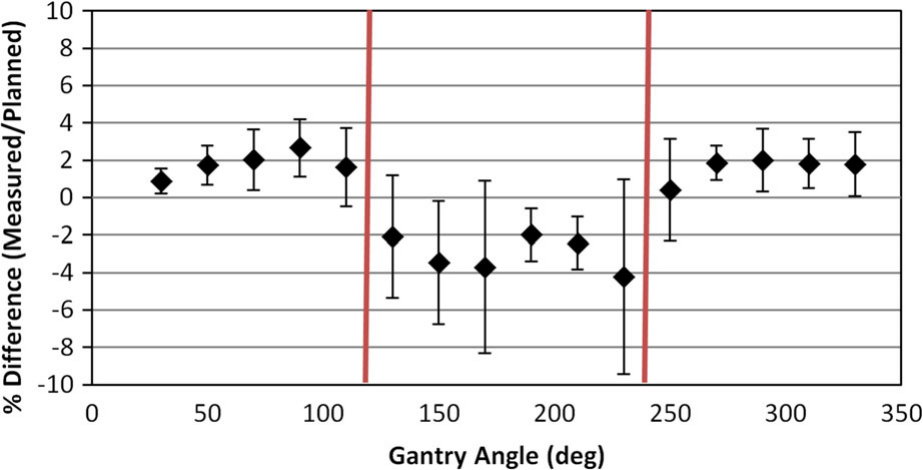
Discrepancy between measured and planned dose as a function of gantry angle with 20° binning. The red vertical lines show the approximate gantry angles where treatment fields begin traversing the phantom support apparatus.

Composite absolute dose results for the nine clinical cases showed a percentage difference from treatment planning system prediction of −2.8% to +1.1% with a mean percentage difference of −0.4% (Table 1). On a per‐field level the mean discrepancy was −0.5% while the mean discrepancy *magnitude* was 2.8%. Studies characterizing the scintillator detector used in this work have found an overall uncertainty between 1.0% and 1.7%.^11^ The larger dose discrepancies between calculated and measured dose are thus not simply due to accuracy of the measurement device, and likely originate from a combination of (a) treatment planning system algorithm accuracy and (b) accuracy of detector placement for these very small fields. Figure 4 plots the per‐field absolute dose agreement as a function of gantry angle. At gantry angles of approximately 120° and 240°, the treatment fields begin to pass through the support apparatus of the BRUNO phantom (baseplate and couch extension). These angles are marked with red lines in Fig. 4, showing that they separate the approximately 2% agreement of the anterior fields from the −2% to −4% agreement of more posterior fields. Our point dose measurements using the scintillation detector matched the total planned dose better than those of Wen et al.^21^ using a pinpoint ion chamber, who found deviations of approximately ±4% for very small targets. The small overall volume and near‐tissue equivalence of the scintillation detector may be advantageous in this regard.

Although the support structures were included in the calculation surface model of the phantom within the iPlan software, this seems to indicate an opportunity for improved support density modeling. Any such improvements to modeling of patient supports will be particular the treatment planning system being used, and it is incumbent on individual institutions to evaluate their particular setup. Another possibility is that the CBCT‐based rotational corrections applied to the BRUNO phantom prior to measurement resulted in an inaccurate geometric representation of the support structures within the plan. That is, the 6D corrections brought the phantom geometry into alignment with the plan but resulted in the support structures no longer having the plan‐assumed geometry. Treatment fields which were calculated to traverse the support structures may not have done so (and vice versa) or they may have traversed a shorter or longer path‐length through those structures. In practice, we attempt to minimize the use of beams that pass through the patient support structures for this reason.

#### 3.B.2 3D dose distribution verification

The isocenter dose point on each of the orthogonal films measured on average within 2.7 ± 0.7% the planned dose of approximately 500 cGy. The FWHM measured 7.2 ± 1.0 mm in the left/right direction, 10.7 ± 0.4 mm in the anterior/posterior direction, and 11.1 ± 1.5 mm in the superior/inferior direction. These values agreed well with the FWHM from the planned dose distributions of 7.1 ± 0.8 mm, 10.8 ± 0.6 mm and 11.0 ± 1.6 mm, respectively (Table 2). For individual films, no systematic difference in the size of the FWHM was observed (0.0 ± 0.2 mm), with the average magnitude of this difference being 0.2 ± 0.1 mm.

**Table 2:** Summary of the film measurement analysis. Planned and measured FWHM of the dose distribution along each of the three principal axes, measured through the position of the 2D dose maximum in each of the three orthogonal planes. Maximum dose in each plane as well as gamma passing rates are also presented

	Plan	Meas.
FWHM (mm)		
Lt./rt.	7.1 ± 0.8	7.2 ± 1.0
Ant./post.	10.8 ± 0.6	10.7 ± 0.4
Sup./inf.	11.0 ± 1.6	11.1 ± 1.5
Max dose (cGy)	497 ± 1	484 ± 4
2D Gamma	92 ± 2% @ 3%/1 mm	

**Figure 5:**
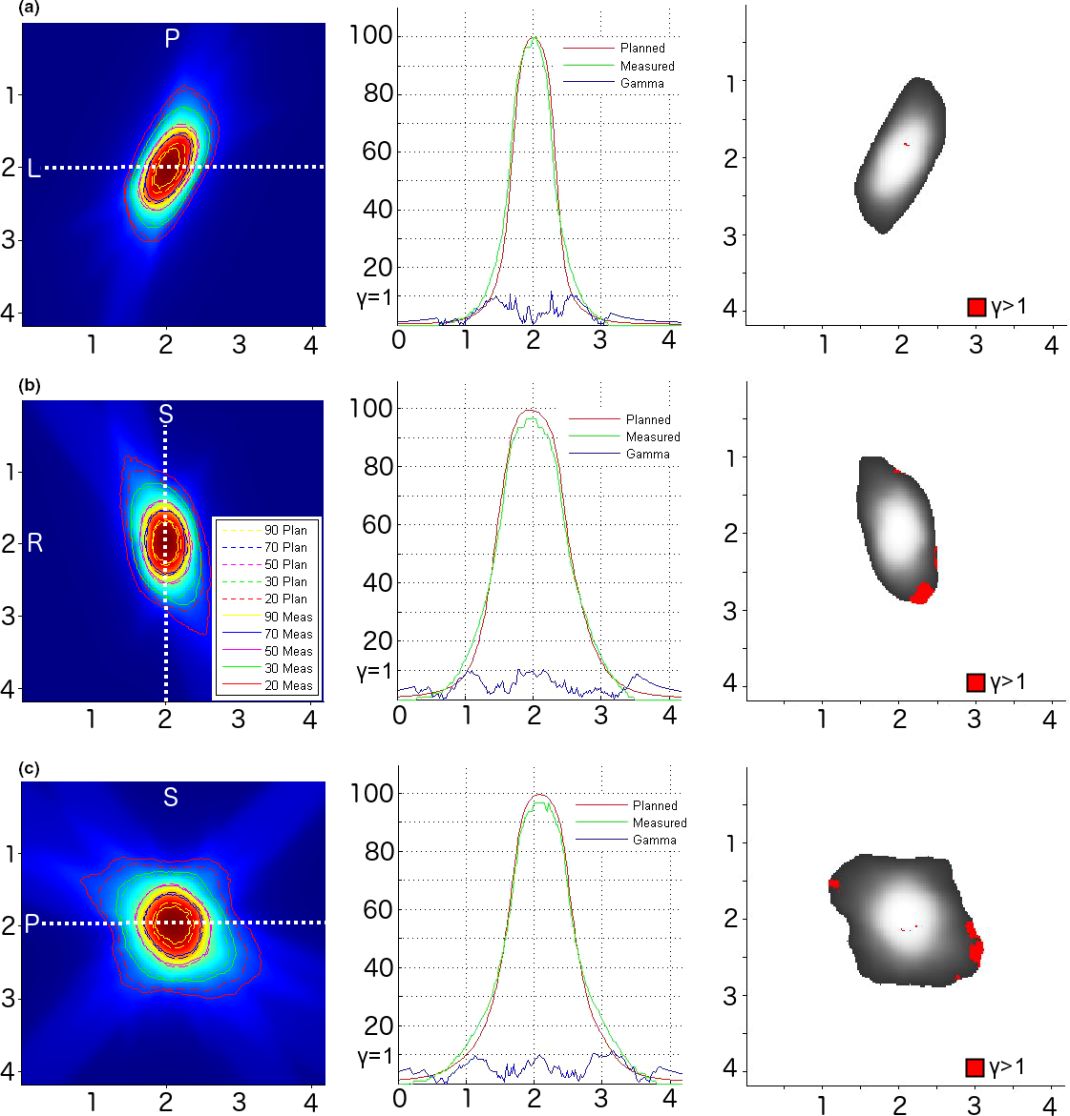
Representative case from four plans measured with film. Left: Measured and planned isodose lines overlaid on the three orthogonal dose planes exported from the treatment planning system: (a) axial, (b) coronal, and (c) sagittal. Center: 1D profiles extracted along each principal axis through the dose maximum (shown by white dotted lines), including the (a) left–right, (b) superior–inferior, and (c) anterior–posterior directions. The 1D gamma analysis is displayed on a ×10 scale for visualization. Right: the 2D gamma analysis pass/fail images (3%/1 mm passing criteria) corresponding to the dose planes on the left‐hand side.

Measured and planned isodoses, dose profiles, and gamma analyses for one representative film are shown in Fig. 5. The largest difference tended to occur either at the field center or at the outer edges of the penumbra between the 10% and 30% isodose lines. The tendency to measure cold near the isocenter and hot in the penumbral regions suggests that a small degree of blurring of the dose distribution due to residual setup error may be responsible. A second possibility is small differences in the position of the film insert from the time of simulation to treatment. The uncertainty in the position of the film cube insert within the BRUNO phantom was measured using digital calipers to be 0.1–0.2 mm depending on the orientation of the insert. The ExacTrac x‐ray verification positioning tolerance of 0.5 mm and 0.5° likely represents an upper limit for the positioning uncertainty of the film.

2D gamma analysis was performed using a 3%/1 mm passing criteria (Fig. 5). A low dose threshold of 20% was employed. This level was chosen as it is similar to the isodose level that would be used to constrain the brainstem for these plans clinically. The average passing rate was 92 ± 2%, comparable to the 95 ± 5% MLC‐based delivery result of Wen et al.^21^ using much larger PTV sizes (0.04–64.4 cc in the CNS). For comparison, using a more conventional low dose cutoff of 10%, the passing rate was 85 ± 2%. It is worth noting that the measured dose distribution was consistently wider than the planned distribution around the 20% isodose level. While the difference was less than 1 mm in most cases, this could impact sparing of the brainstem. This level of uncertainty should thus be taken into account during the planning process, for example through a planning risk volume expansion, controlling the proximity of the target and brainstem structures, or employing a lower brainstem dose planning goal.

Two common delivery techniques for trigeminal neuralgia are linac with collimating cones and Gamma Knife. Our film results are comparable to the investigation of cone‐based SRS by Wiant et al.,^22^ which found a difference between planned dose distributions and film measurements of up to 0.3 mm in terms of FWHM, and an absolute dose agreement no worse than 3.6%. Somigliana et al.^23^ performed film and diode measurements for the Gamma Knife treatment unit, and also found a similar FWHM agreement for the smallest collimating helmet (4 mm) of 0.1–0.2 mm. In their study, the output factor for the 4 mm collimators differed by 6% for film measurements and 10% for diode measurements when compared to the preconfigured planning system, although a later recommendation to increase the output factor for this collimator by 9% would largely eliminate these discrepancies.^24^ Finally, a gamma analysis of film dosimetry for the Gamma Knife by Park et al.^25^ found 100% agreement at 1%/1 mm using a low dose threshold of 20%, both for single 4 mm apertures and for a composite plan using 4, 8, and 16 mm fields. The ability to achieve such high gamma passing rates with Gamma Knife in phantom is likely due to the absence of rotating couch or gantry parts on this treatment unit. Overall, comparable treatment delivery accuracy was achieved with the HDMLC‐based linac to cone‐based delivery or Gamma Knife, with the exception being seen in film gamma analysis where Gamma Knife excels. Image‐ or frame‐based realignment at each couch position is prudent for linac‐based deliveries (both HDMLC and cones) to reduce the blurring effect of intrafraction motion on dose distribution.

The validation results from this study are in line with historical trends for SRS of intracranial targets. The Lutz et al.^2^ study first established the methodology of precise targeting of a cranial SRS tumor from CT localization using a linac system equipped with circular collimating cones. They showed that an idealized target (a small radiopaque ball) imaged with CT and a stereotactic localization frame could be irradiated within a positional accuracy of 1.3 ± 0.6 mm in any direction when positioned with stereotactic frame to the room lasers. In modern linac systems equipped with kV imaging panels, stereotactic alignment has improved with the aid of online CBCT image guidance.^26^ Huang et al.^7^ evaluated the overall positioning accuracy of image‐guided intracranial radiosurgery across multiple linear accelerator platforms. They demonstrated that submillimeter accuracy on the order of 0.5 mm could be achieved for residual setup errors using ExacTrac on the Novalis or CBCT on the TrueBeam platforms. They determined these residual setup accuracies from the portal images of a ball bearing target in a 2 × 2 cm^2^ treatment field defined by the MLC in the manner of Lutz et al., but did not attempt to show the dose localization accuracy with respect to isocenter placement and beam shaping. Chang et al.^27^ investigated the clinically relevant accuracy of CyberKnife radiosurgery system using a head phantom containing a large spherical target of 32 mm diameter and CyberKnife's circular collimator delivery system under kV image guidance. They showed that for a large target and circular beam collimation (fixed), the mean placement of the prescription isodose cloud is within 1.1 ± 0.3 mm. Our results with HDMLC beam shaping for much smaller target sizes and isocenter dose cloud placement with CBCT guidance compare well with this work.

## 4 CONCLUSIONS

Deliverability of clinical trigeminal neuralgia plans utilizing HD‐MLC was validated using scintillator‐based absolute dose measurements, radiochromic film measurements of 3D spatial dose deposition, and log‐file‐based assessment of the positioning accuracy of individual MLC leaves. The results demonstrated an ability to achieve absolute dosimetric accuracy of better than 3% utilizing both scintillator and film measurement techniques, along with FWHM agreement between measured and planned dose profiles within 0.2 mm in each principal delivery plane. Inspection of trajectory log files revealed a maximum MLC leaf positioning error of 0.04 mm across nine clinical cases. In addition to following published small field treatment planning recommendations,^28^ the tests outlined in this study could be used by an institution looking to commission a program to treat trigeminal neuralgia with HD‐MLC.

In conjunction with a quality‐controlled treatment delivery methodology, an appropriately commissioned treatment planning system can be used for accurate and clinically appropriate design of trigeminal neuralgia treatment plans utilizing HD‐MLC.

## CONFLICT OF INTEREST

No conflicts of interest to disclose.
